# Optimizing woodcutting with zirconia-toughened alumina: Processing, performance, and industrial insights

**DOI:** 10.1016/j.heliyon.2025.e41785

**Published:** 2025-01-08

**Authors:** Tamanna Thakur, Stefan Heinen, Bruno Ehrle, Gurdial Blugan

**Affiliations:** a Swiss Federal Laboratories for Materials Science and Technology (Empa), Laboratory for High Performance Ceramics, 8600, Dübendorf, CH, Switzerland; b OERTLI Werkzeuge AG, Hoftstrasse 1, 8181, Höri bei Bülach, Switzerland

**Keywords:** Ceramics, Wood cutting, Zirconia, Alumina, Sintering

## Abstract

Since the 1950s, the woodcutting industry has relied heavily on tungsten carbide (WC) cutting tools to overcome the challenges posed by the complex structure of wood, including hard knots and abrasive elements such as sand and tannic acids. These demands require cutting tools with superior thermal conductivity and mechanical properties. However, the rising cost of WC materials has prompted the search for alternative solutions. As a result, zirconia-toughened alumina (ZTA) ceramic materials with varying amounts of in situ formed SrAl_12_O_19_ have been introduced as potential substitutes. This study focuses on the processing, microstructural characterization, and mechanical behavior of these ceramic cutting tools with the goal of matching or exceeding the cutting performance and tool life of conventional WC tools. The study demonstrates the effectiveness of the improved ceramic tools through numerical evidence obtained from short-term trials and subsequent extended high-speed tests conducted on industrial cutting machines. In particular, comparable wood surface quality and wear resistance were achieved along with a significant improvement in cutting speed, resulting in a threefold reduction in machining time. These results underscore the potential of ceramic cutting tools as a cost-effective and efficient alternative in the woodcutting industry.

## Highlights

• Zirconia-toughened alumina ceramics with SrAl_12_O_19_ offer a cost-effective alternative to WC cutting tools.
• Adding 2.5 wt% SrAl_12_O_19_ optimized tool performance, matching or exceeding WC tools in woodcutting.
• Ceramic tools cut solid beech three times faster than traditional WC blades.
• Finer grain size in the ceramic powders improved hardness and cutting efficiency, boosting tool performance.
• ZTA ceramic blades cut costs, reduced processing time, and increased productivity in woodcutting.

## Graphical abstract

**Figure:**
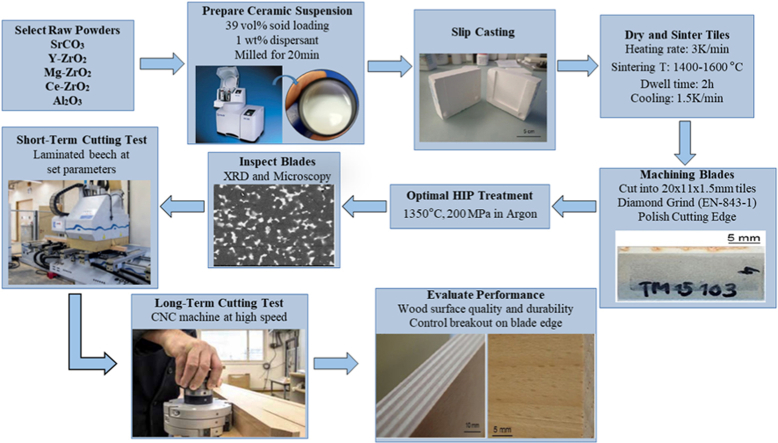

## 1 Introduction

Wood is a challenging material to cut due to its variable hardness and inhomogeneous composition, including knots and fibers. This complexity places high mechanical demands on cutting tools [1,2]. Liquid coolants can damage wood, so blades must have good thermal conductivity. Achieving smooth wood surfaces for industrial use is essential and requires rigorous testing of new ceramic tool compositions for both short-term and high-speed industrial cutting tests. The cutting behavior of wood is influenced by its structure, which varies with wood species and growth conditions. The tannic acid content of wood, which is affected by moisture, can chemically erode tungsten (WC) cutting blades [3,4]. Industrial wood cutting speeds vary from 40 m/s for hard woods to 60 m/s for soft woods, and blades must withstand temperatures up to 800 °C [5]. Blades typically consist of a steel holder that often holds multiple blades. Lighter blades allow for higher rotational and cutting speeds, improving woodworking efficiency. Surface finish is critical in the wood industry and depends on blade quality, workpiece homogeneity and cutting variables. Beech, with fewer knots, is commonly used for cutting tests [[6], [7], [8]]. Cutting tools require high performance materials with specific properties such as strength, hardness, toughness, thermal stability, conductivity and low affinity to the workpiece material [[9], [10], [11]]. Common materials include high speed steel, cermet, diamond, cubic boron nitride, cemented carbides, and cermets such as WC with a cobalt binder [7,12]. Tungsten is not found in its natural state and is primarily extracted from the minerals wolframite and scheelite, with Russia, Canada, and China accounting for 80 % of global production. The growing demand for tungsten, fueled by its exceptional properties and diverse applications, has contributed to this price increase. Additionally, the high density of tungsten carbide (WC) restricts the rotational speed of cutting tools (mechanical properties of WC are detailed in Table S1). These challenges underscore the need for alternative materials that can deliver similar performance while enabling higher cutting speeds, greater productivity, and a more stable supply of raw materials such as non-oxide ceramics, particularly silicon carbide (SiC) and silicon nitride (Si_3_N_4_) [[13], [14], [15], [16]]. A previous study focused on improving the performance of cutting materials using an Si_3_N_4_ base infused with silicon carbide (SiC) to improve fracture toughness and thermal conductivity [13,17]. These hot-pressed Si_3_N_4_/SiC tools lasted three times longer than tungsten carbide tools, but production costs were high [18]. To address this, a near-net-shape process was developed to simplify sintering, while additional additives such as MgO, Al_2_O_3_, and Y_2_O_3_ were explored to promote densification and improve mechanical properties [[19], [20], [21], [22]]. It is noteworthy that oxide ceramics exhibit superior machinability compared to Si_3_N_4_/SiC composites [23]. In Addition, oxide ceramics are easier to manufacture. Given their lower raw material costs, this could make the industrial production of ceramic inserts more economical. Recent studies have shown that the incorporation of submicron Al_2_O_3_ particles into the composition of cutting tools improves their mechanical properties and edge durability [24,25]. Guo et al. [26] have shown that the introduction of ZrO_2_ into Al_2_O_3_-based cutting materials results in superior tribological properties compared to tools composed of Al_2_O_3_, Si_3_N_4_, or WC alone. This improvement is attributed to ZrO_2_'s ability to inhibit chemical reactions, thereby increasing frictional resistance [27]. However, Sommer et al. [28,29] have focused attention on the injection molding of Al_2_O_3_ and zirconia toughened alumina (ZTA) ceramics, demonstrating that the presence of residual voids and low toughness contribute significantly to chipping during machining of medium-density fiberboard, resulting in inferior surface quality compared to tungsten carbide tools. Gogolewski et al. [30] and Beer et al. [24] have investigated submicron Al_2_O_3_ and Al_2_O_3_-ZrO_2_ ceramics with different grain sizes and identified chipping and abrasive wear as two predominant failure mechanisms, concluding that their unpredictable failure modes make them unsuitable for industrial use.

Additionally, Al_2_O_3_ demonstrates the ability to form ternary oxides with various alkali, alkaline earth, and rare earth oxides, adopting the magnetoplumbite structure by crystallizing into hexagonal platelets. The inclusion of these platelets enhances the overall toughness of the ceramic material. While particles, whiskers, or fibers are commonly used as reinforcement materials, platelets can also activate toughening mechanisms effectively. Platelets can be introduced either by directly adding them or by using chemical compounds that form platelets in situ during sintering [31,32]. In this study, strontium carbonate (SrCO_3_) was added to form strontium hexaluminate (SrAl_12_O_19_ or SrO·6Al_2_O_3_), which has a needle-like morphology [33]. SrAl_12_O_19_ has shown promise in enhancing the fracture toughness (K_Ic_) of Al_2_O_3_-based ceramics. However, research on both the intrinsic properties of SrAl_12_O_19_ and its role as a reinforcement in ceramic composites remains limited.

Strontium (Sr) serves as a viable alternative for platelet formation, with SrCO_3_ acting as the precursor to produce SrAl_12_O_19_ according to the reaction:

(1) SrCO_3_ + 6 ∙Al_2_O_3_ → SrAl_12_O_19_+ CO_2_

The magnetoplumbite structure of SrAl_12_O_19_ promotes the growth of hexagonal platelets during sintering. The high-temperature conditions facilitate platelet nucleation and growth, which significantly influence the composite's microstructure. This in situ reaction simplifies the manufacturing process by eliminating the need for pre-synthesized SrAl_12_O_19_ and ensures a uniform distribution of platelets throughout the ceramic matrix. Achieving the desired microstructure and mechanical properties requires optimizing both sintering conditions and precursor ratios [34].

Previous research has demonstrated the effectiveness of additives like Y_2_O_3_, CeO_2_, MgO, CaO, and TiO_2_ in enhancing the properties of zirconia, including increasing its critical diameter, stabilizing the tetragonal phase at room temperature, and making zirconia-toughened alumina (ZTA) composites suitable for use in cutting tools and wear-resistant applications [[35], [36], [37], [38], [39], [40]]. Y_2_O_3_ is the most commonly used stabilizer for zirconia (ZrO_2_) and serves as a foundational material. Studies have explored two types of Y-TZP with different grain sizes [28]. Adding CeO_2_ improves the mechanical properties of ZTA up to a point; however, excessive CeO_2_ content causes crystallization that forms a magnetoplumbite structure CeAl_11_O_18_ similar to SrAl_12_O_19_, which can degrade the material's performance [41,42]. The optimal CeO_2_ content for stabilizing ZrO_2_ is between 10 and 12 mol%. Cutler et al. described ceria-stabilized ATZ as a material for plough tips, which could withstand high stresses without fracturing [43]. On the other hand, adding MgO to ZTA reduces hardness due to the formation of spinel. However, it's unclear whether higher hardness directly improves cutting performance, which is why the impact of Y-TZP, nanoscale Y-TZP, Mg-PSZ, and Ce-TZP on cutting performance is being studied.

This work focuses on the processing, microstructural characterization and mechanical behavior of zirconia-toughened alumina (ZTA) prepared using different alumina powders and zirconia with different stabilizers with the addition of different amounts of in situ formed SrAl_12_O_19_ for industrial cutting tools. Since there is no relevant mechanical or material test that can accurately characterize or predict the cutting performance, the blades of each developed composition were first tested in a short term cutting test and successful compositions were subjected to a longer-term high-speed test using industrial cutting machines to validate their cutting performance. In addition, the effect of ZTA sintering methods, including spark plasma sintering (SPS), on cutting performance was investigated.

## 2 Experimental

### 2.1 Materials

The starting materials used in this work are strontium sarbonate (SrCO_3_), two kinds of yttrium stabilized zirconia (Y-ZrO_2_), partially stabilized zirconia (Mg-PSZ), Ce-ZrO_2_, and three kinds of alpha alumina (α-Al_2_O_3_) listed in Table 1. The densities of all dried powders were measured with a Helium pycnometer (AccuPyc II 1340, Mircomerities, USA) and humidity of the as received powders was measured using a moisture analyser (Mettler Toledo HR83 Halogen, Switzerland). The particle size distribution was analyzed by laser light scattering (Beckman Coulter LS 230 Small Volume Module Plus, USA). The surfaces of all as received powders were also investigated using a secondary electron microscope (SEM, VEGA TS 5136 MM, TESCAN, Czech Republic) to see the morphology and presence of agglomerates.

**Table 1:** Summary of Materials used, Trade Names, Companies and their properties.

Compound	Trade Name	Company	Density (g/cm^3^)	Humidity (%)	d_50_ (μm)
SrCO_3_	Strontium Carbonate	Sigma Aldrich	3.7	0.22	2.850
Y-ZrO_2_	TZ-3YS-E	Tosoh	5.98	0.21	0.432
Y-ZrO_2_	TZ-3Y-E	Tosoh	5.83	0.36	0.200
Mg-PSZ	PMD-a	Auer Remy	5.71	0.08	1.089
Ce-ZrO_2_	–	EMPA	6.14	0.02	–
*α*-Al_2_O_3_	Nabalox NO 710-13	Nabaltec	3.98	0.82	0.377
*α*-Al_2_O_3_	TM-DAR	Taimicron	3.95	0.22	0.158
*α*-Al_2_O_3_	CT3000LS-SG	Alcoa	3.93	0.39	0.387

### 2.2 Ceramic suspensions and characterization

Four experimental series were conducted. In Series I, the initial composition consisted of 80 wt% Al_2_O_3_ and 20 wt% ZrO_2_ (3Y-TZP), was selected based on established literature highlighting the mechanical and tribological advantages of ZTA composites, serving as the reference formulation for this study [44]. The first series focused on the influence of varying SrAl_12_O_19_ content on the cutting performance by forming in situ theoretical 2.5 wt%, 5.0 wt% and 7.5 wt% SrAl_12_O_19_ [31,32]. The compositions from this series are listed in Table 2. The second series examined the effect of different Al_2_O_3_ powders, particularly their particle sizes. The composition is shown in Table 3. The third series investigated various types of stabilized Zirconia. In this series, Y-TZP is substituted with nanoscale Y-TZP (nY-TZP), Ce-TZP, and Mg-PSZ, while maintaining a fixed SrAl_12_O_19_ content of 2.5 wt% and utilizing alumina TM DAR from Taimicron. Additionally, the effect of incorporating 10 wt% and 20 wt% of Y-TZP is assessed in samples containing nanoscale Y-TZP. It is important to note that the fracture toughness of ZTA reaches an optimum at a ZrO_2_ content between 10 and 12 wt% [45]. For the compositions involving Ce-TZP and Mg-PSZ, a ZrO_2_/Al_2_O_3_ ratio of 10/90 was selected to minimize agglomerate formation due to the larger particle sizes. The compositions for this series are detailed in Table 4. In fourth series, selected samples from the previous three series to undergo a post-machining thermal stress-relaxing treatment.

**Table 2:** Summary of compositions in Series I.

Sample name	Wt%			Density (g/cm^3^)	Sintering T [°C]
	Al_2_O_3_	ZrO_2_	SrAl_12_O_19_		
	Nabalox NO 710-13	TZ-3YS-E			
ZTA(N) 0 SrA	80	20	0	4.26	1600
ZTA(N) 2.5 SrA	80	20	2.5	4.26	1600
ZTA(N) 5.0 SrA	80	20	5.0	4.25	1600
ZTA(N) 7.5 SrA	80	20	7.5	4.24	1600

**Table 3:** Summary of compositions in Series II with corresponding wt% of Al_2_O_3_, ZrO_2_, and SrAl_12_O_19_, as well as sintering temperatures T in °C.

Sample name	Wt%				Density (g/cm^3^)	Sintering T [°C]
	Al_2_O_3_		ZrO_2_	SrAl_12_O_19_		
	TM DAR	CT3000 LS-SG	TZ-3YS-E	–		
ZTA(T) 2.5 SrA	80	–	20	2.5	4.26	1500
ZTA(T) 10/90 2.5 SrA	90	–	10	2.5	4.11	1400
ZTA(C) 2.5 SrA 1600	80	–	20	2.5	4.26	1600
ZTA(C) 2.5 SrA 1550	80	–	20	2.5	4.26	1550
ZTA(TC) 2.5 SrA 1550	40	40	20	2.5	4.26	1550
ZTA(TC) 2.5 SrA 1500	40	40	20	2.5	4.26	1500

**Table 4:** Summary of compositions in Series III with according wt% of Al_2_O_3_, ZrO_2_, and SrAl_12_O_19_, as well as sintering temperatures T in °C.

Sample name	Wt%					Density (g/cm^3^)	Sintering T [°C]
	Al_2_O_3_		ZrO_2_		SrAl_12_O_19_		
	TM DAR	TZ-3Y-E	Ce-TZP	Mg-PSZ	–		
n-ZTA(T) 2.5 SrA	80	20	–	–	2.5	4.26	1450
n-ZTA(T) 10/90 2.5 SrA	90	10	–	–	2.5	4.11	1450
Ce-ZTA(T) 10/90 2.5 SrA	90	–	10	–	2.5	4.11	1475
Mg-ZTA(T) 10/90 2.5 SrA	90	–	–	10	2.5	4.11	1500

**Fig. 1:**
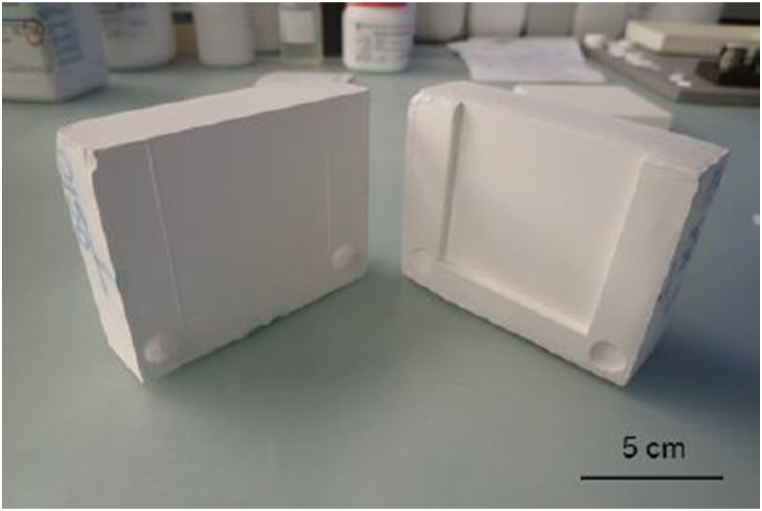
Plaster molds with dimensions: 67 × 67 × 5 mm.

Aqueous suspensions for slip casting were prepared with 39 vol% solid loading and 1 wt% dispersant (Dolapix PC 75, Zschimmer & Schwarz GmbH, Lahnstein, Germany). These suspensions were milled in a planetary mill for 20 min (PM 400/4, Retsch GmbH, Germany) and then poured into gypsum plaster molds of 67 × 67 × 5 mm, as shown in Fig. 1 [46]. The plaster contact on both large surfaces ensured uniform water removal, preventing tile curvature after sintering. Drying of the molds was also carried out at ambient temperature until a constant weight was achieved. Sintering was performed in a Carbolite GmbH HTF 1800 furnace, Germany. The heating rate was set to 3 K/min up to the sintering temperature, followed by a 2 h dwell time in air, and cooling at the 1.5 K/min down to room temperature. Rheological properties of the suspensions were examined with the Anton Paar rheometer MCR 302, equipped with a coaxial cylinder geometry, measuring viscosity across a shear rate range of 0.1–100 s^−1^ at a constant temperature of 25 °C.

### 2.3 Machining of blanks/blades and characterization

After sintering all tiles were ground and cut into 20 × 11 × 1.5 mm blanks and diamond ground in accordance to standard EN 843-18. Machined blanks of series I were HIPed (Hot Isostatic Pressing) at 1350 °C and blades of series II and III at 1300 °C and 200 MPa for 2 h in argon. Densities after sintering and HIPing were measured with Archimedes' method. Subsequently, the cutting edge of the blade was machined on the blanks by Oertli AG with a target radius of <5 μm. Before accepting the blades for the cutting trials, optical light microscopy (Discovery V20, Carl Zeiss Jena, Germany) was used to identify eventual outbreaks. X-ray diffraction (XRD) analysis was performed on sintered samples for phase identification with the equipment PANalytical X'Pert Pro MPD (Netherlands equipped with Johansson mono-chromator, Cu-Kα,λ = 1.5405 Å). The measurements were carried out for 2θ values between 10° and 80° for 60 min. The data has been processed by X'Pert HighScore Plus.

For Vickers hardness measurement (HV), the ceramic surface was polished to 1 μm using a diamond suspension (MetaDi®, Buehler, USA). Hardness was measured according to EN 843-4 using a Durimet micro hardness indenter (Ernst Leitz, Wetzlar, Germany) with a 2 kp load for 15 s. Five indentations were made, and diagonal lengths were measured with an integrated light microscope.

Young's modulus (E) was measured using the Impulse Excitation Method in accordance with DIN EN 843-2. The testing was performed with a Grindo-Sonic Mk5 “Industrial” instrument (J.W. Lemmens, Belgium). During the test, a light hammer strike was applied to the test bar, and a detector positioned beneath it recorded the natural frequency of vibration.

### 2.4 Wood cutting tests

#### 2.4.1 Short term cutting tests

The short-term cutting tests were designed to quickly assess the cutting performance of various blade compositions. These tests were performed on a milling machine (type HAC, GF Brugg, Switzerland) with an assisted feed system (Holzher, Switzerland). Laminated beech wood was chosen for its unique challenges, such as the presence of glue joints and two distinct fiber orientations (parallel and perpendicular), which allowed for the early identification of potential weaknesses in the blades. These tests were conducted with moderate cutting speeds and lengths to focus on initial wear behavior and surface quality, minimizing variables and providing a rapid evaluation of blade performance. The cutting parameters (Table S2) used in these trials included a cutting length of approximately 3.75 m, a cutting depth of 5 mm, a speed of 56.5 m/s, a rotation speed of 6,000 rpm, and a feed rate of 8 m/min. Following the tests, the wood surfaces were carefully examined for abnormalities, and the best-performing blades were selected for further evaluation in long-term cutting trials.

#### 2.4.2 Long term cutting tests

In contrast, the long-term cutting tests aimed to evaluate the durability and sustained performance of the most promising blades under conditions more representative of industrial applications. The tests were carried out with a computerized numerical control (CNC) machine (Venture 7L and Optimat BHC 550, Weeke, Germany). These trials were conducted with knot-free solid beech wood, ensuring consistent material properties across a total cutting length of 1,000 m. The cutting speeds and durations were significantly increased to simulate high-speed, continuous operation typical in industrial settings. Cutting parameters (Table S2) for the long-term tests included a cutting depth of 1 mm, a cutting speed of 150.5 m/s, a rotation speed of 20,100 rpm, and a feed rate of 30 m/min. After every 250 m of cutting, the wood surface was inspected, and the cutting edges of the blades were examined under optical light microscopy. This allowed for detailed evaluation of any mechanical integrity issues, such as edge outbreaks, which could compromise performance.

By incorporating both short-term and long-term trials, this study effectively screens blade compositions for potential issues while rigorously validating their performance under realistic, high-stress industrial conditions. This dual approach ensures that the blades not only perform well in the short term but also maintain reliability and durability for extended periods, offering a comprehensive evaluation of their cutting effectiveness.

## 3 Results & discussion

The starting materials were characterized using particle size analysis, density, humidity and SEM imaging. The densities and humidity values of all the powders used are presented in Table 1. Fig. S1 shows the particle size distribution curve and SEM image of SrCO_3_ powder, where some needle-like grains exceed 5 μm in length and have diameters below 0.5 μm. The needle-like shape of SrCO_3_ particles complicates particle size analysis, as light scattering techniques assume spherical particles for accurate measurements [47]. The particle size distributions and SEM images of all Al_2_O_3_ samples are provided in Fig. S2. Among them, Al_2_O_3_ TM DAR from Taimicron has the smallest primary particle size and is the only one with a monomodal distribution, with particles being nearly spherical due to their co-precipitation production process (Fig. S2d). Nabalox NO 710-13 and CT3000 LS-SG have similar distributions; however, their particles, derived from larger fragments, are not spherical (Figs. S2b and c). The TZ-3YS-E and TZ-3Y-E powders, produced via a hydrolysis process, result in spherical particles (Figs. S3b and c). TZ-3YS-E has a d_50_ of 0.432 μm and a monomodal distribution, while TZ-3Y-E has smaller particles prone to agglomeration, leading to polymodal peaks in the distribution due to the measurement of agglomerates rather than primary particle size (Fig. S3c). The SEM image of ceria-stabilized ZrO_2_ powder reveals small, angular particles, with a few brighter ones suggesting incomplete reaction with ceria (Fig. S3d). Mg-PSZ, delivered with a particle size of <5 μm, shows the widest range of particle distribution. However, the particles are broken and crushed during production (Fig. S3e), affecting the particle size measurement depending on how they interact with the laser in the Beckman Coulter equipment. Although some particles are up to ∼5 μm in size, literature suggests that this particle size may be too large for optimal cutting performance [24,30].

**Fig. 2:**
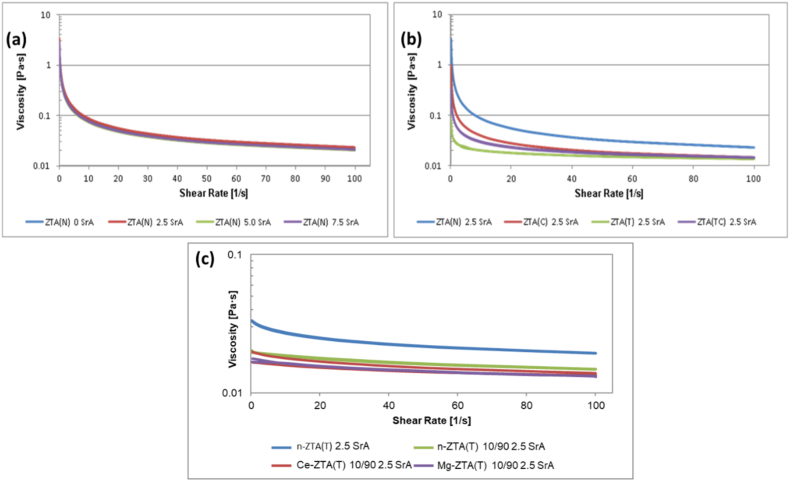
Viscosity curves of casting slips of (a) series I, (b) series II, and (c) series III.

Fig. 2 illustrates the viscosities of the casting slips for Series I, II, and III. In Series I, all casting slips exhibit shear-thinning behavior without any thixotropy. In Series II, the ZTA(T) 2.5 SrA suspension with TM DAR has a significantly lower viscosity compared to the other suspensions. This is attributed to the much smaller and more spherical Al_2_O_3_ particles, which improve the wetting of individual particles by water and dispersant. The viscosity of the ZTA(TC) 2.5 SrA suspension with mixed Al_2_O_3_ falls between that of the TM DAR and CT3000LS-SG suspensions, as expected. Series III displays similar behavior, with the Ce-ZTA(T) 10/90 2.5 SrA and Mg-ZTA(T) 10/90 2.5 SrA suspensions showing less thixotropy. However, all suspensions remain within a viscosity range suitable for slip casting.

### 3.1 Microstructure of cutting blades

**Fig. 3:**
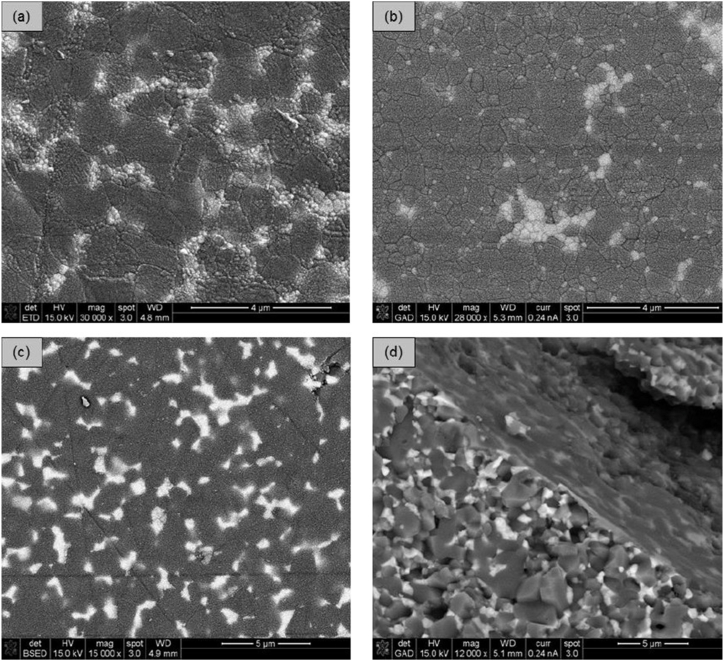
SEM of (a) etched surface of ZTA(T) 10/90 2.5 SrA, (b) etched surface of n-ZTA(T) 10/90 2.5 SrA, (c) etched and (d) fracture surface of ZTA(N) 0 SrA (white grains = ZrO_2_).

**Fig. 4:**
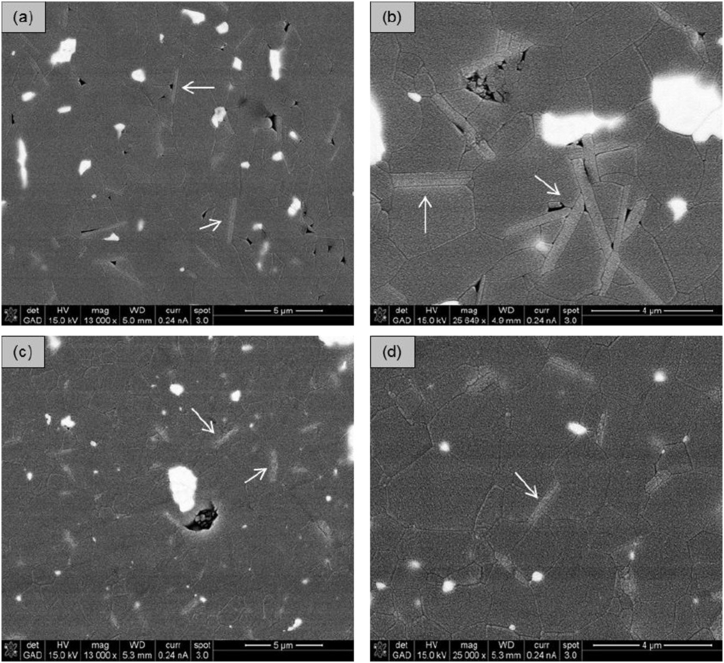
BS-SEM of etched surfaces of (a,b) Mg-ZTA(T) 10/90 2.5 SrA and (c,d) Ce-ZTA(T) 10/90 2.5 SrA (Arrows mark needle like shapes of SrAl_12_O_19_).

**Fig. 5:**
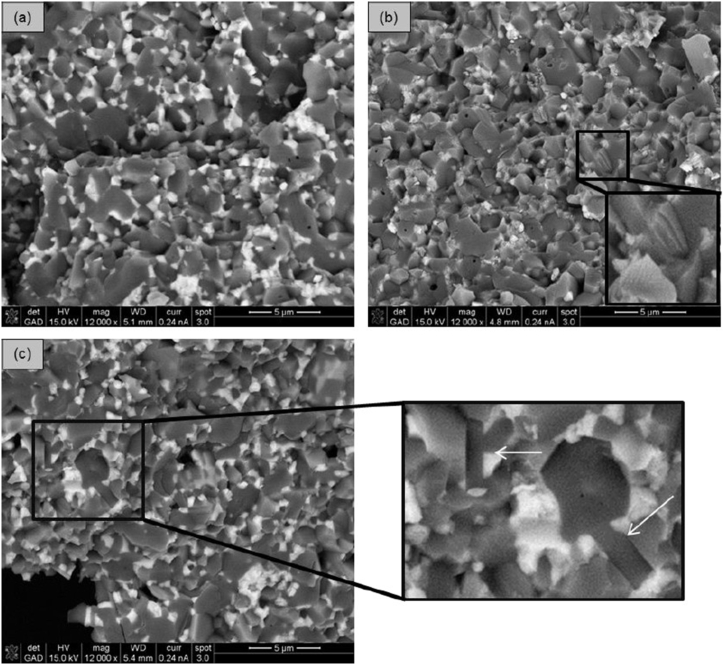
Fracture surfaces of (a) ZTA(N) 0 SrA (reference), (b) ZTA(N) 2.5 SrA and (c) ZTA(N) 7.5 SrA with SrAl_12_O_19_ needles.

The microstructures of the dense ceramics were examined on polished and thermally etched surfaces using scanning electron microscopy (SEM). Fig. 3a–c displays thermally etched surfaces, while Fig. 3d shows a smooth surface without thermal etching. The etched surfaces reveal widely distributed ZrO_2_ grains (appearing white) within an Al_2_O_3_ matrix (appearing grey), along with agglomerates of ZrO_2_ grains, suggesting that further optimization of the suspension homogenization is possible. Generally, compositions with a ZrO_2_/Al_2_O_3_ ratio of 10/90 exhibit more ZrO_2_ agglomerates than those with a 20/80 ratio. Additionally, nanoscaled Y-TZP forms more agglomerates than Y-TZP due to smaller grain sizes and stronger attractive forces within the agglomerates. SrAl_12_O_19_ particles exhibit a needle-like shape, with lengths of less than 3 μm and widths of approximately 0.5 μm. The quantity of visible SrAl_12_O_19_ needles varies across most samples. Ce-ZTA(T) 10/90 2.5 SrA and Mg-ZTA(T) 10/90 2.5 SrA show a high concentration of SrAl_12_O_19_ needles (Fig. 4). Higher sintering temperatures promote particle growth, making SrAl_12_O_19_ more prominent in these compositions. Both samples display open porosity, indicating suboptimal sintering. The large average grain sizes and porosity of Mg-PSZ and Ce-TZP are likely to negatively impact the cutting performance of the blades. Fracture surfaces were also analyzed to detect SrAl_12_O_19_. Fig. 5a illustrates the microstructure of 20/80 ZTA from the reference material (ZTA(N) 0 SrA), where ZrO_2_ grains are uniformly distributed within the Al_2_O_3_ matrix. ZTA(N) 2.5 SrA exhibits a similar fracture surface structure, though SrAl_12_O_19_ needles are less visible. Additionally, some Al_2_O_3_ grains display cleavage steps, indicating grain fracture. XRD measurements for all three series were conducted and are provided in the SI section 2.

### 3.2 Mechanical properties of materials

**Fig. 6:**
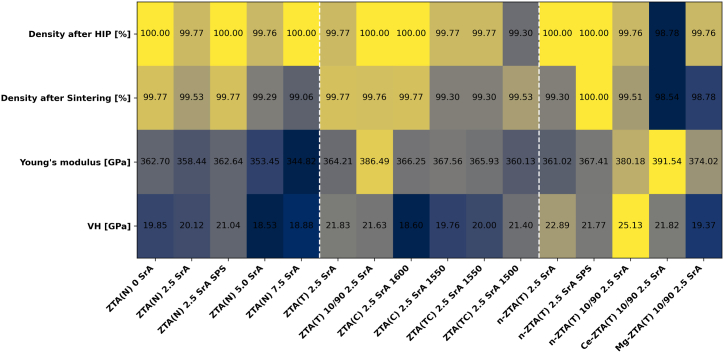
Heat map showing mechanical properties: density after HIP (%), density after sintering (%), Young's modulus (GPa), and Vicker's Hardness (GPa) for Series I, II, and II, separated with dashed lines, color code represents gradients for each row from low (blue), over medium (grey), to high (yellow) values.

For all compositions, four key properties were measured: density after HIP, density after sintering, Young's modulus, and Vickers hardness. Fig. 6 presents a summary of the mechanical properties for all compositions across series I-III. Sintering achieved high densification, reaching over 99% of the theoretical density (mentioned in Table 2, Table 3, Table 4), resulting in low porosity and enhanced mechanical edge integrity of the cutting blades. Post-HIPing, density further increased, as shown in Fig. 6 (first two rows), with the process closing any remaining open porosity. This densification improves cutting edge integrity by relieving stress after surface grinding. Additionally, densities after HIPing that were lower than the reference, such as in ZTA(T) 2.5 SrA and ZTA(TC) 2.5 SrA at 1550 °C, should be interpreted cautiously. Notably, the exceptions, Ce-ZTA(T) 10/90 2.5 SrA and Mg-ZTA(T) 10/90 2.5 SrA, are attributed to the specific sintering behavior of those particular ZrO_2_s within the ZTA composites. Moreover, a comparison with tungsten carbide (WC) cutting blades (see Table S1) highlights a key advantage of these ceramic wood-cutting blades. Although WC is renowned for its exceptional hardness and durability, its high density imposes limitations on the rotational speed of modern cutting tool systems. In contrast, the ceramic blades, with their significantly lower density, allow for higher rotational speeds, enhancing cutting performance while maintaining excellent mechanical stability. This combination of lightness and structural integrity offers a more efficient and precise cutting experience, making the ceramic blades a superior alternative in high-speed applications.

The Vickers hardness of compositions in Series I shows consistent values, suggesting that the presence of in-situ formed SrAl_12_O_19_ has minimal impact on hardness. Both ZTA(N) 0 SrA and ZTA(N) 2.5 SrA exhibit similar hardness levels, but further addition of SrCO_3_, leading to increased in-situ SrAl_12_O_19_ formation, results in a hardness reduction of approximately 1.50 GPa. Therefore, 2.5 wt% in-situ SrAl_12_O_19_ appears to be the optimal amount. Notably, ZTA(N) 2.5 SrA sintered by SPS demonstrates higher hardness compared to conventional sintering, attributed to the smaller grain sizes achieved through the rapid sintering process. In Series II, comparing samples with the same amount of in-situ formed SrAl_12_O_19_ but different Al_2_O_3_ types reveals that using TM DAR Al_2_O_3_ yields higher hardness due to its smaller grain size and higher purity. However, there is no significant difference in hardness (only 0.2 GPa) between samples made with 80 wt% and 90 wt% TM DAR, with this variation falling within the data's margin of error. Samples incorporating CT3000LS-SG and a mixture of CT3000LS-SG with TM DAR exhibit the highest hardness at slightly lower sintering temperatures. In Series III, the incorporation of nano-ZrO_2_ in ZTA results in higher hardness compared to ZTA(T) 2.5 SrA with the same Al_2_O_3_ and a ZrO_2_/Al_2_O_3_ ratio of 20/80. The highest hardness of 25.13 ± 0.93 GPa was observed in the n-ZTA(T) 10/90 2.5 SrA samples, followed by n-ZTA(T) 2.5 SrA with 80 wt% Al_2_O_3_. Surprisingly, similar compositions sintered by SPS exhibit lower hardness. As expected, the sample with Mg-stabilized ZrO_2_ shows the lowest hardness in Series III, due to the larger initial grain size of ZrO_2_ and the inherently lower hardness of Mg-PSZ. Furthermore, the ceramic wood-cutting blades exhibited greater hardness than WC (Table S1), enhancing wear resistance and edge retention for precise, long-lasting cuts. This makes them ideal for high-speed woodworking, offering superior durability and performance compared to traditional WC tools.

For Series I, Young's modulus decreases as the content of in situ formed SrAl_12_O_19_ increases. Additionally, the purity of Al_2_O_3_ appears to have a minimal effect on Young's modulus, as similar values are observed in Series II. In contrast, increasing the Al_2_O_3_ content while maintaining a constant amount of SrAl_12_O_19_ results in a higher Young's modulus due to the inherently higher modulus of Al_2_O_3_. In Series III, the ZTA(T) 10/90 2.5 SrA composition exhibits a higher modulus than the same composition with a smaller grain size of ZrO_2_ (n-ZTA(T) 10/90 2.5 SrA). Moreover, ceria stabilization leads to the highest Young's modulus, measured at 391.54 ± 1.99 GPa.

The ceramic sintered parts exhibited a lower Young's modulus than tungsten carbide (WC), indicating that WC is stiffer but also more brittle, making it prone to chipping under dynamic loads. In contrast, ceramic blades, with their lower modulus, achieve an optimal balance between hardness and toughness, providing superior resilience, edge retention, and performance in high-speed wood-cutting applications.

### 3.3 Short term cutting tests

**Fig. 7:**
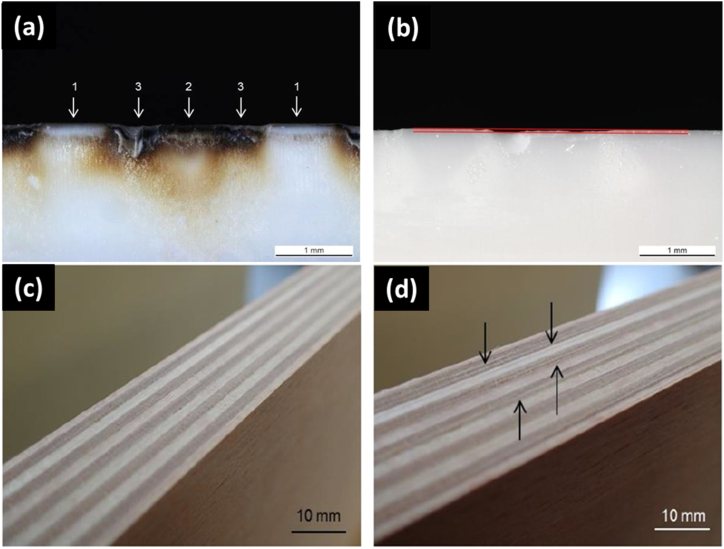
Images of (a) a cutting edge with glue and wood residue after short term cutting test, (b) cleaned cutting edge with lines which mark the smallest outbreak and cut wood in short term cutting tests showing (c) excellent and (d) bad surfaces.

For the short-term cutting trials, laminated beech was selected due to the high abrasiveness of its glue joints. It was hypothesized that if the blade performed well on laminated beech during these trials, it would likely endure the long-term cutting trials. Fig. 7a shows a cutting blade after a short cutting test, displaying a typical wear pattern typical wear pattern: 1 section cut along fibre direction (parallel), 2 section cut across fibre direction (perpendicular) and 3 glue joint. All blade compositions were tested in the short-term trials, with certain compositions undergoing additional heat treatment (HT) before testing, labeled as series IV. Following the short-term cutting trials, the blades were examined using optical microscopy. The heat generated during cutting caused the glue from the laminated beech to melt, adhere to the blades, and harden. To remove this residue, the blades were heated to 1000 °C in air for 2 h to burn off the glue and wood residues. Fig. 7b shows a typical example of the largest breakout observed. Two types of breakouts were identified: small ones, which could cause damage to the cut wood surface, and larger breakouts consisting of several chips joined together, typically associated with the glue and areas where the fibers were perpendicular.

In addition to the depth of outbreaks caused by the cutting blades, the quality of the cut wood surface is of paramount importance. Unacceptable wood surfaces are characterized by significant outbreaks (quality grade 5.0), pronounced cross-sectional profiles, and pulled fibers in the longitudinal section. Pulled fibers that are barely noticeable to the touch against the cutting direction, resulting in a certain degree of surface roughness, are considered acceptable (quality grades between 2.0 and 5.0). Excellent wood surfaces (quality grades between 1.0 and 2.0) are smooth and exhibit no variations in the cross-section of the laminated wood. Fig. 7 shows images of cut wood in short term cutting tests showing excellent (c) and bad (d) surfaces.

**Fig. 8:**
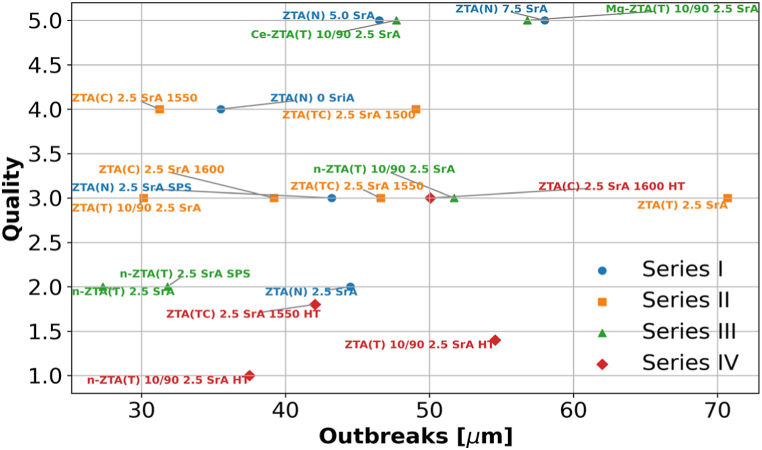
Quality vs Outbreaks for all series (I-IV) in short term tests showing best performing series as IV.

Cutting tests were conducted on 20 different blade compositions, with two blades per composition evaluated. The average outbreak depth and surface quality results are summarized in Fig. 8. Blades that underwent post-heat treatment (HT) demonstrated superior wood surface quality and cutting performance overall. Specifically, the n-ZTA(T) 10/90 2.5 SrA HT blade achieved the smoothest surface, followed by the ZTA(T) 10/90 2.5 SrA HT and ZTA(TC) 2.5 SrA 1550 HT blades, as depicted in Fig. 7c. The ZTA(C) 2.5 SrA 1600 HT blade also performed similarly to the best non-heat-treated blade, n-ZTA(T) 2.5 SrA. Most blade compositions produced acceptable wood surfaces; however, those containing 5.0 and 7.5 wt% SrAl_12_O_19_, as well as Ce- and Mg-stabilized ZrO_2_, exhibited the poorest surface quality. Blades with outbreak depths exceeding 50 μm generally resulted in a decline in wood cut quality from excellent to acceptable or unacceptable, except for the ZTA(T) 10/90 SrA HT blade. The ZTA(TC) 2.5 SrA blades sintered at both temperatures, Ce-ZTA(T) 10/90 2.5 SrA, and Mg-ZTA(T) 10/90 2.5 SrA had comparably large outbreaks. Nonetheless, the ZTA(TC) 2.5 SrA blades produced notably better wood surfaces compared to the Ce-ZTA(T) 10/90 SrA blades, as shown in Fig. 7d. This indicates that wood surface quality does not have a straightforward correlation with the depth of outbreaks in the cutting edge. In Series I (Fig. 8), an increase in SrAl_12_O_19_ content was associated with larger outbreaks, suggesting that an optimal SrAl_12_O_19_ content is 2.5 wt% for achieving the desired transformation from SrCO_3_ to SrAl_12_O_19_. Series II results indicated that smaller grain sizes in cutting blades led to better cutting performance, both in terms of outbreak depth and wood surface quality. In Series III, n-ZTA(T)-stabilized compositions outperformed others, and a clearer correlation between outbreak depth and cutting quality was observed. Additionally, the impact of cutting speed was assessed for three compositions: ZTA(N) 2.5 SrA, ZTA(T) 10/90 2.5 SrA HT, and n-ZTA(T) 10/90 2.5 HT. New cutting parameters were set as: cutting depth of 2 mm, rotation speed of 20,100 min⁻^1^, feed rate of 30 m/min, and cutting speed of 150.5 m/s for high-speed tests. As anticipated, higher cutting speeds negatively affected wood quality, reducing it by one grade but still within the acceptable range (≤3). Detailed results and parameters are provided in the supplementary information, (Section 3 Cutting Speed).

### 3.4 Long term cutting tests

**Fig. 9:**
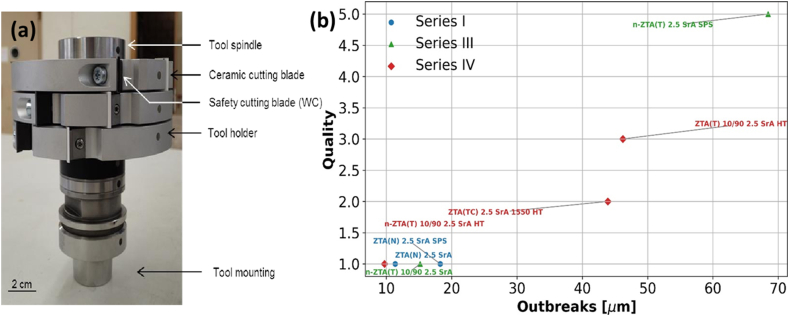
(a) Tool spindle with three tool holders used for long term cutting test, (b) Quality vs Outbreaks for a selected number of compositions in three different series.

**Fig. 10:**
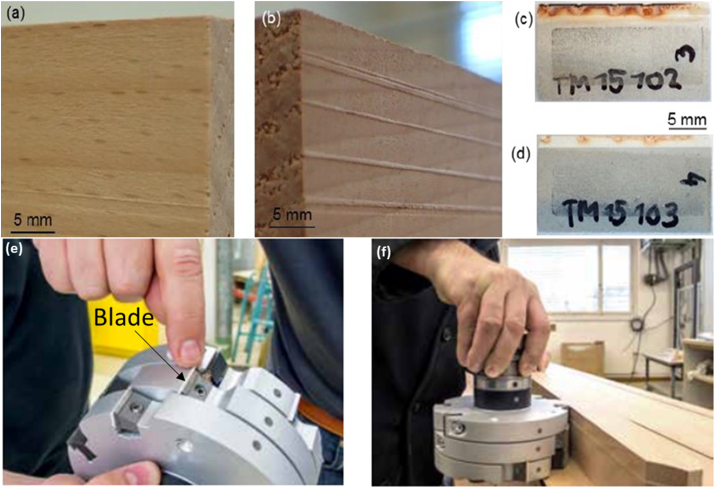
(a) Excellent wood surface cut by n-ZTA(T) 10/90 2.5 SrA HT and (b) unacceptable wood surface cut by n-ZTA(T) 2.5 SrA SPS, Blade 1 of n-ZTA(T) 2.5 SrA SPS (c) heavily worked and (d) blade 2 lightly worked during long term cutting test, (e) tool spindle with ceramic blade and (f) wood cutting process.

Seven different compositions were selected for long-term cutting trials based on the quality of wood surfaces observed in short-term tests. Fig. 9a displays the tool spindle used in these long-term tests, while Fig. 9b highlights the largest outbreaks and the quality scores of the cutting blades post-trial. Among the tested compositions, n-ZTA(T) 2.5 SrA SPS exhibited the deepest outbreaks and near-regular patterns along the cutting edge, resulting in an unacceptable wood surface. However, the quality of the surface remained consistent throughout the entire cutting process, as shown in Fig. 10b. This anomaly is likely due to the fact that only one blade was actively machining the wood, while the other blade engaged only in the areas affected by the first blade's outbreaks, as depicted in Fig. 10b–d. Unlike in the short-term trials, the depth of the outbreaks in long-term tests appears to be directly correlated with the quality of the wood surface (Fig. 9b). All compositions, except for n-ZTA(T) 2.5 SrA SPS, delivered acceptable to excellent results in the wood surface tests. The quality of the wood surface is closely linked to the size of the outbreaks on the cutting edge, with optimal surfaces being produced when outbreaks are less than 20 μm. The compositions ZTA(N) 2.5 SrA, ZTA(N) 2.5 SrA SPS, and n-ZTA(T) 10/90 2.5 SrA, with or without post-heat treatment, performed the best, as shown in Fig. 10a. These compositions not only achieved excellent surface quality but also allowed for machining at speeds three times faster than conventional WC blades, increasing from 40 to 60 m/s to 150 m/s [5,13]. Fig. 10e and f shows the tool spindle equipped with a ceramic blade and the wood cutting process in action.

## 4 Conclusion

This research demonstrates the potential of zirconia-toughened alumina (ZTA) cutting blades with in situ formed SrAl_12_O_19_ as a transformative solution for the woodcutting industry, offering a viable alternative to conventional tungsten carbide (WC) tools. Comprehensive evaluations of 20 distinct compositions, incorporating short-term and extended high-speed cutting trials, revealed key insights into material composition and processing strategies. The findings underscore the significance of optimizing SrAl_12_O_19_ content, with 2.5 wt% identified as the optimal level to enhance fracture toughness and minimize mechanical edge degradation. Fine-grained alumina powders significantly improved mechanical properties such as hardness and young's modulus, enabling superior cutting-edge retention. Furthermore, the incorporation of nanoscale yttria-stabilized zirconia (nY-TZP) enhanced the homogeneity of the microstructure, facilitating a balance between hardness and toughness essential for high-speed woodcutting applications. The cutting blades fabricated with compositions such as n-ZTA(T) 10/90 2.5 SrA HT not only exhibited excellent wood surface finishes in short-term trials but also maintained durability and performance in long-term industrial tests. Notably, these ceramic tools achieved cutting speeds up to 150 m/s, three times faster than standard WC blades, while delivering comparable or superior wood surface quality. This efficiency leap presents significant industrial advantages, including reduced machining time and enhanced operational throughput. Additionally, the study highlights the critical role of advanced sintering techniques such as spark plasma sintering (SPS) in achieving superior microstructural properties, particularly for compositions with nanoscale ZrO_2_. However, the trade-offs between sintering methods and resultant properties, such as reduced hardness in SPS-treated samples, warrant further investigation. By leveraging lightweight and thermally stable ceramic composites, this work paves the way for a paradigm shift in industrial woodcutting, emphasizing not only the technical superiority of ZTA tools but also their potential for economic and sustainable manufacturing processes. Future research should focus on scaling production techniques and exploring hybrid compositions to further refine the balance of mechanical properties, ensuring broader applicability across diverse woodcutting conditions and materials.

## CRediT authorship contribution statement

**Tamanna Thakur:** Writing – review & editing, Writing – original draft, Validation, Methodology, Investigation, Formal analysis, Data curation. **Stefan Heinen:** Writing – original draft, Visualization, Software, Formal analysis, Data curation. **Bruno Ehrle:** Validation, Resources, Investigation. **Gurdial Blugan:** Writing – review & editing, Validation, Supervision, Resources, Methodology, Investigation, Conceptualization.

## Declaration of competing interest

The authors declare that they have no known competing financial interests or personal relationships that could have appeared to influence the work reported in this paper.
