# Supplementary material for: Optimizing woodcutting with zirconia-toughened alumina: Processing, performance, and industrial insights
Source: Heliyon. 2025 Jan 8;11(2):e41785. doi: 10.1016/j.heliyon.2025.e41785 (PMC11774802; doi:10.1016/j.heliyon.2025.e41785)
Supplement: Multimedia component 1 [file mmc1.docx]

Supplementary Information

**Optimizing Woodcutting with Zirconia-Toughened Alumina: Processing, Performance, and Industrial Insights**

Stefan Heinen^a*^, Tamanna Thakur^a^, Bruno Ehrle^b^, Gurdial Blugan^a^

*^a^Swiss Federal Laboratories for Materials Science and Technology (Empa) Laboratory for High Performance Ceramics 8600 Dübendorf CH*

*^b^OERTLI Werkzeuge AG, Hoftstrasse 1, 8181 Höri bei Bülach, Switzerland*

# Table S1: Mechanical Properties of WC [6]

| Property | Unit | WC |
| --- | --- | --- |
| Density | g/cm^3^ | 15.7 |
| Tensile strength | MPa | 1550 |
| Young's Modulus | GPa | 720 |
| Fracture Toughness | MPa m^1/2^ | <10 |
| Hardness | GPa | 20.8 |

# Characterization of Starting Materials

**
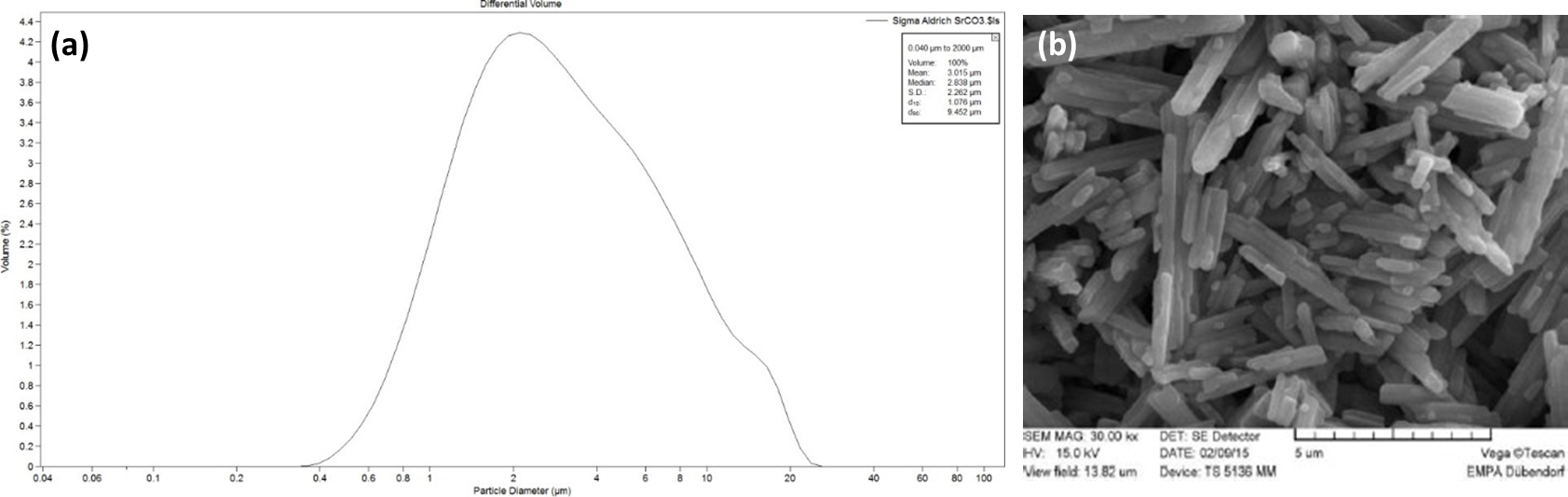
**

**Figure S 1:** (a) Particle size distribution and (b) SEM of SrCO_3_ (Sigma Aldrich)


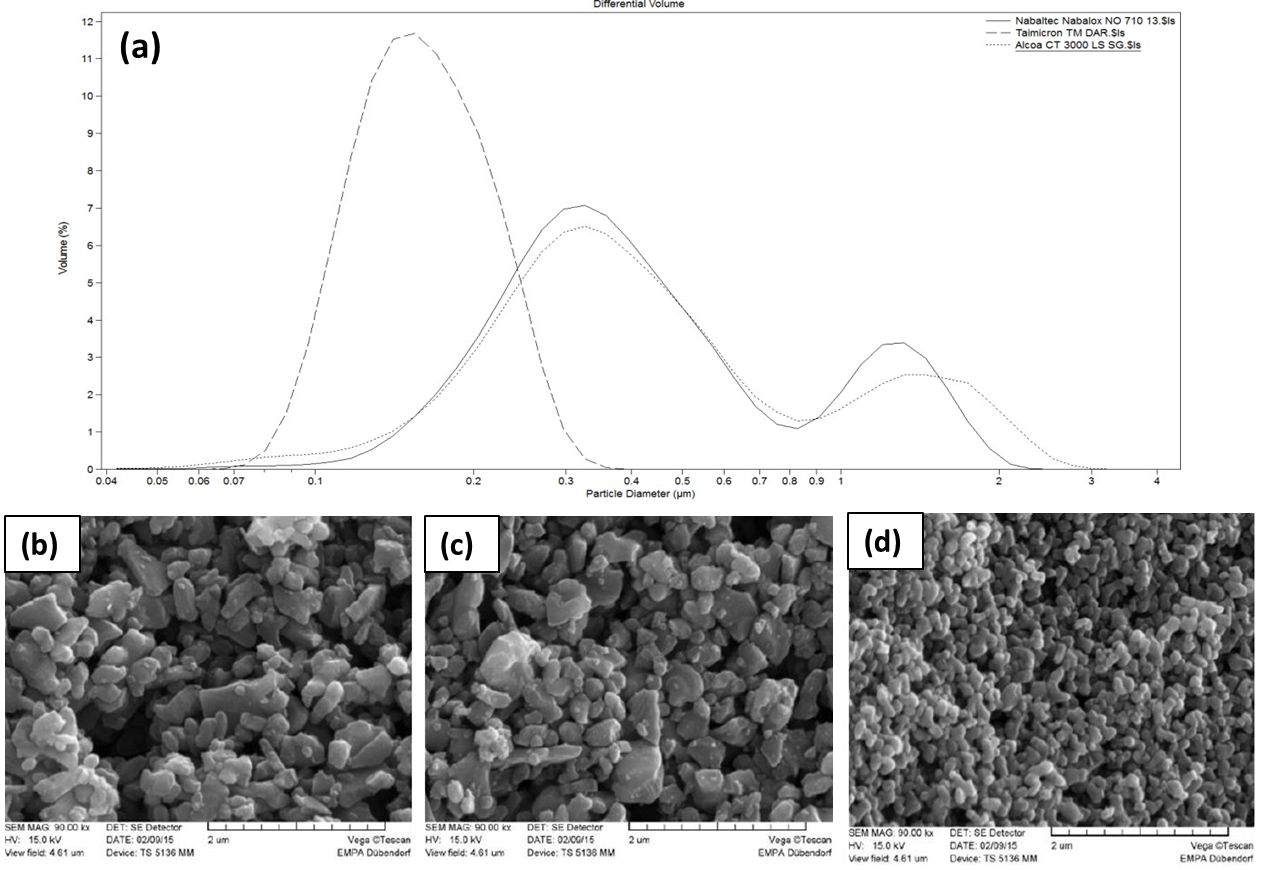


**Figure S 2:** (a) Particle size distribution and SEM of (b) Nabalox NO 710-13 (Nabaltec), (c) TM DAR (Taimicron) and (d) CT3000LS-SG (Alcoa)


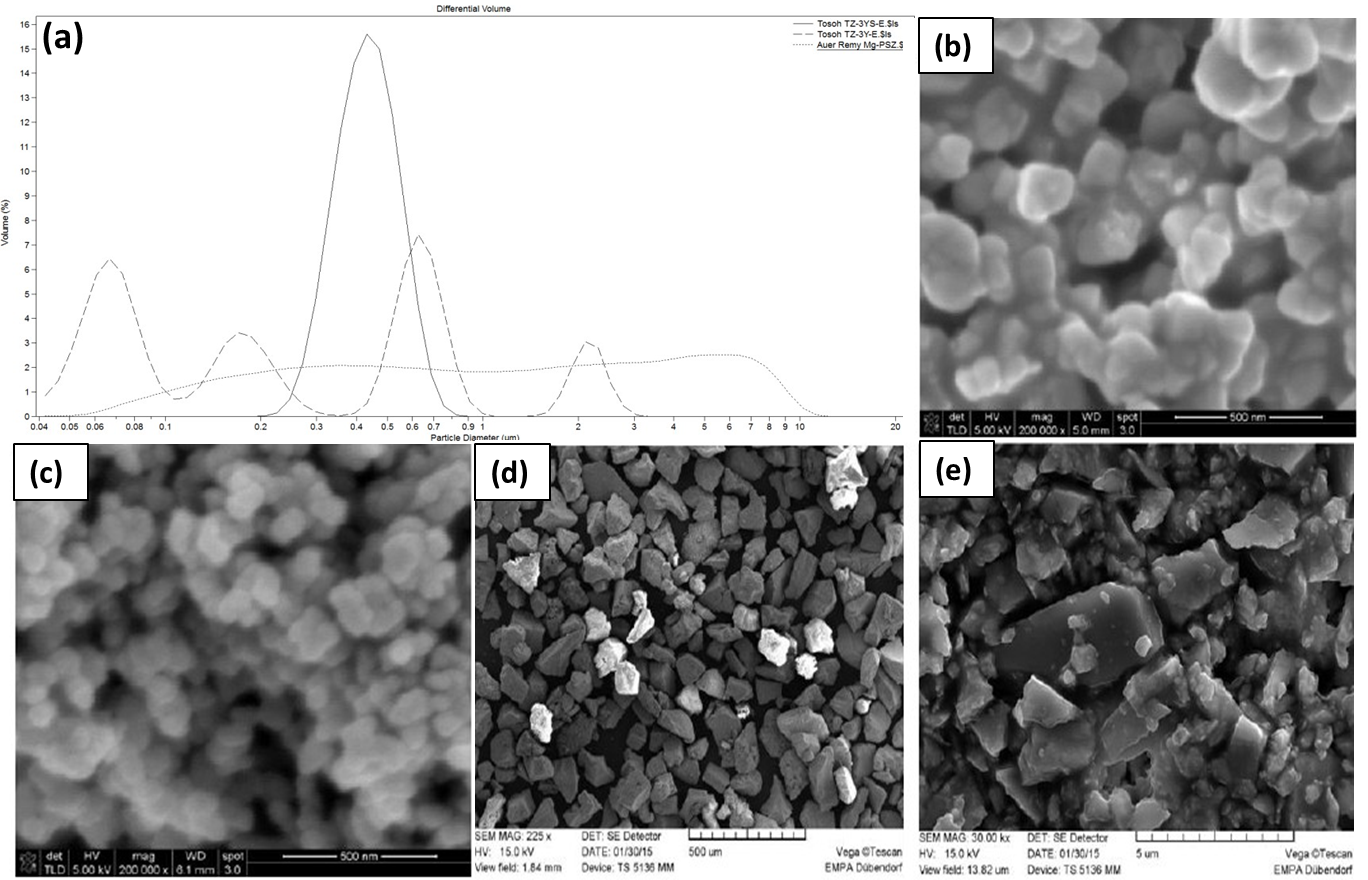


**Figure S 3:** (a) Particle size distribution and SEM of (b) TZ-3YS-E (Tosoh), (c) TZ-3Y-E (Tosoh) (c) Ce-TZP (Empa) and (d) Mg-PSZ (Auer Remy)

# X-ray diffraction (XRD) analysis

The XRD patterns for Series I are presented in Figure S4. During sintering, SrAl_12_O_19_ was formed in all samples doped with SrCO_3_. No other Sr-containing compounds were detected, suggesting that no additional phases were formed, except in the case of ZTA(N) 2.5 SrA, which was sintered using SPS. In this particular sample, a second phase of strontium aluminate (SrAl_2_O_4_) was observed. It is likely that the 2 min dwell time during SPS was insufficient for the complete formation of SrAl_12_O_19_, as compared to the 2 h duration required for conventional sintering. Given the identical chemical composition of Series II, the XRD patterns are similar to those of Series I and shown in Figure S5. The chemical composition of Series III varies depending on the type of ZrO_2_ stabilization, as illustrated in Figure S6. When comparing compositions stabilized with Y_2_O_3_, no differences in phases were observed. Specifically, in the n-ZTA(T) 2.5 SrA sample sintered by SPS, only SrAl_12_O_19_ was formed, whereas in the ZTA(N) 2.5 SrA sample sintered by SPS, both SrAl_12_O_19_ and SrAl_2_O_4_ phases were present.


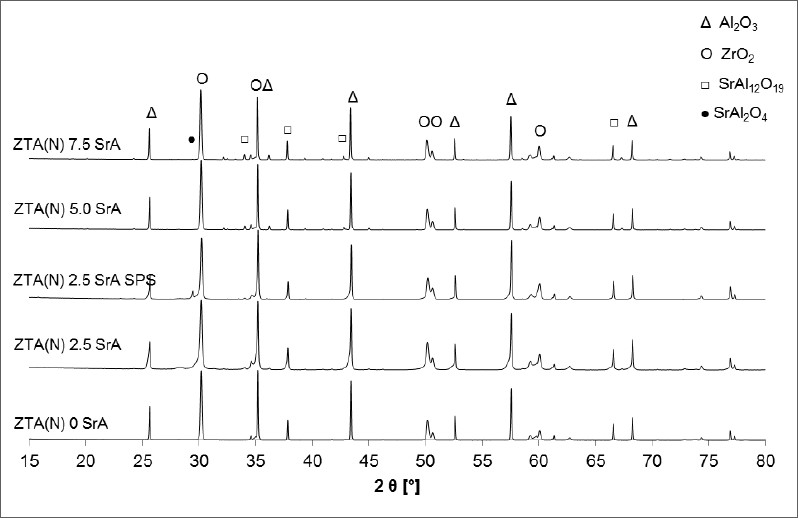


**Figure S4:** XRD pattern for Series I

To optimize the SPS parameters for ZTA(N) 2.5 SrA, it is essential to prevent the formation of SrAl_2_O_4_. Compositions incorporating Ce-TZP and Mg-PSZ in ZTA exhibit lower ZrO_2_ peak intensities compared to Y-TZP. In the case of Ce-ZTA(T) 10/90 2.5 SrA, neither CeO_2_ nor other Ce-based compounds were detected. Conversely, in Mg-ZTA(T) 10/90 2.5 SrA, a secondary magnesia-rich ZrO_2_ phase was observed, with a reduction in the original ZrO2 phase. Specifically, a distinct ZrO_2_ second phase, Mg_0.125_Zr_0.875_O_1.875_, was identified in Mg-ZTA(T) 10/90 2.5 SrA, likely originating from the starting powder phase of Mg_0.2_Zr_0.8_O_1.8_ (Figure S7), which transforms into Mg_0.125_Zr_0.875_O_1.875_ during sintering. However, Mg_0.2_Zr_0.8_O_1.8_, the primary phase in Mg-PSZ, was found to be unsuitable for this application due to the resulting phases and grain sizes in the ZTA composition. Unfortunately, alternative Mg-PSZ materials could not be evaluated due to constraints in time and availability.


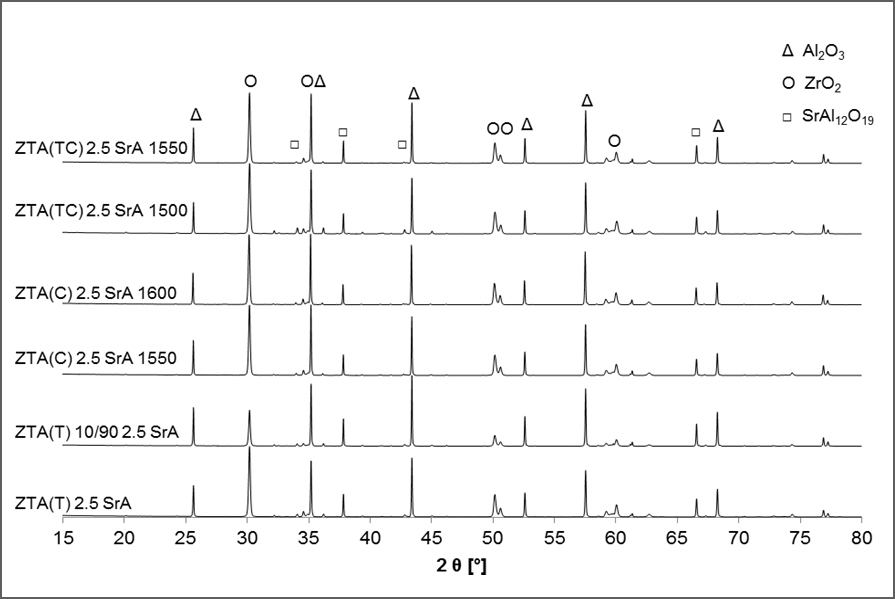


**Figure S5:** XRD pattern for Series II


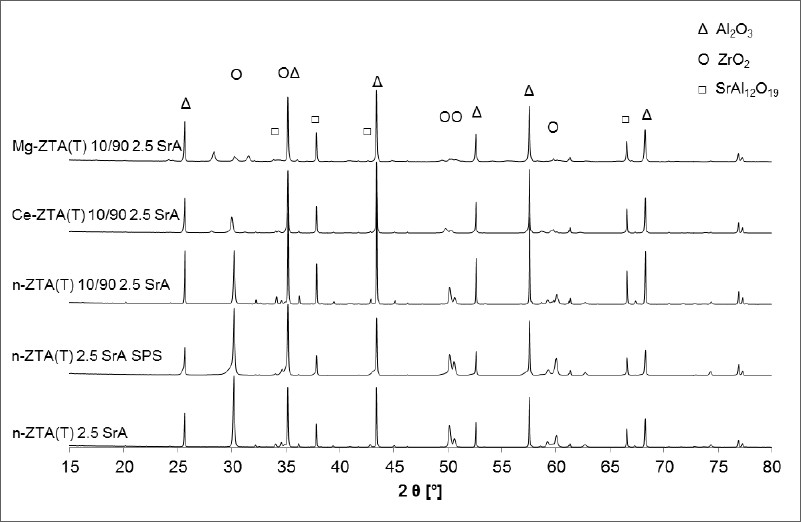


**Figure S6:** XRD pattern for Series III


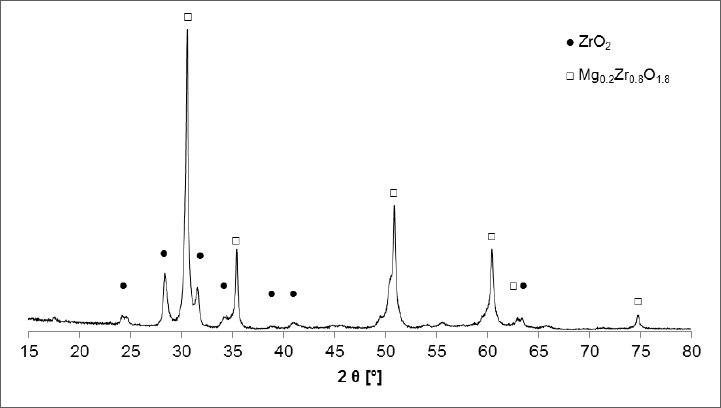


**Figure S7:** XRD of Mg-PSZ powder

# Cutting Speed

**Table S2:** Parameters for cutting process at low and high speeds of laminated beech

| **Parameters** | **Low Speed** | **High Speed** |
| --- | --- | --- |
| Cutting depth (mm) | 5 | 2 |
| Rotation speed (min*^−^*^1^) | 6’000 | 20’100 |
| Feed rate (m/min) | 8 | 30 |
| Cutting speed (m/s) | 56.5 | 150.5 |


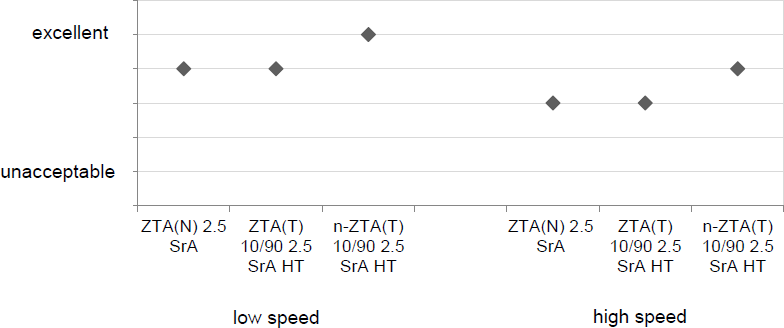


**Figure S8:** Quality of cut wood surface after short term cutting test at low and high speed.
